# Frailty and osteoporotic fractures represent mutual risks for each other with common physiological backgrounds

**DOI:** 10.1093/jbmrpl/ziaf009

**Published:** 2025-01-13

**Authors:** Tomohiko Urano, Tatsuhiko Kuroda, Mitsuru Saito, Masataka Shiraki

**Affiliations:** Department of Geriatric Medicine, School of Medicine, International University of Health and Welfare, Narita City 286-8686, Chiba, Japan; Public Health Research Foundation, Shinjuku-ku 169-0051, Tokyo, Japan; Department of Orthopedic Surgery, School of Medicine, Tokyo Jikei University, Minato-ku 105-8471, Tokyo, Japan; Research institute and Practice for Involutional Diseases, Azumino City 399-8101, Nagano, Japan

**Keywords:** frailty, osteoporosis, osteoporotic fracture, adiponectin, Il-6

## Abstract

Frailty and osteoporosis are known to exacerbate each other. However, limited research is available on the shared pathophysiological factors contributing to osteoporotic fractures and frailty. This study aims to identify common factors associated with both the current frailty and the occurrence of incident vertebral fractures. A total of 912 postmenopausal Japanese women, 63.9 ± 10.0 yr of age (mean ± SD), were included in this study. Each participant’s baseline frailty status was assessed using a questionnaire about the following 5 items: fatigue, resistance, ambulation, inactivity, and weight loss. A score of 3 or above indicated the prevalence of frailty. The participants were then followed up for an average of 10.5 ± 7.5 yr, during which 202 patients suffered incident vertebral fractures. The Cox proportional hazards model for incident vertebral fracture revealed that lumbar bone mineral density (hazard ratio [HR] 0.753, *p*<.001), adiponectin (HR 1.025, *p*=.021), log IL-6 (HR 1.227, *p*=.029), prevalent vertebral fracture (HR 2.124, *p*<.001), and frailty status (HR 1.355, *p*=.002) were independent predictors of incident vertebral fractures. The factors associated with frailty status at baseline were assessed using logistic regression analysis, revealing that adiponectin (odds ratio [OR] 1.063, *p*<.001), log IL-6 (OR 2.94, *p*<.001), and prevalent vertebral fractures (OR 2.816, *p*<.001) were significantly associated with current frailty. Biochemical factors such as IL-6 and adiponectin were commonly associated with vertebral fractures and frailty. Additionally, frailty status was identified as an independent risk factor for vertebral fractures, while prevalent vertebral fractures were significantly associated with frailty. These findings clearly indicate that frailty and osteoporotic fractures represent mutual risks for each other, with serum levels of adiponectin and IL-6 serving as common physiological backgrounds.

## Graphical Abstract

**Graphical Abstract:**
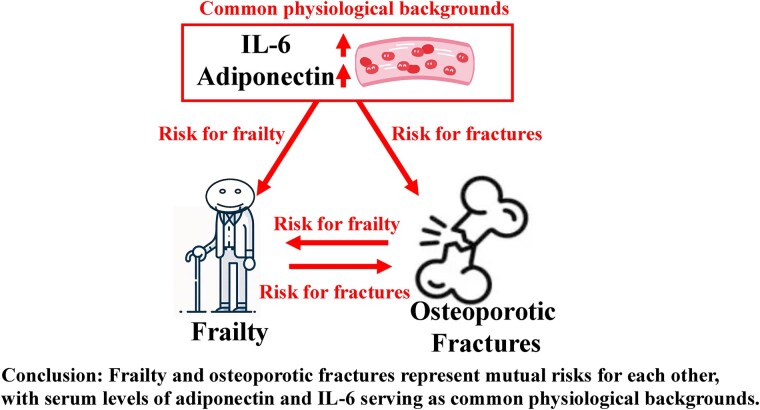

## Introduction

Osteoporosis is a condition characterized by reduced bone strength, leading to an elevated susceptibility to fractures. Fractures arising from osteoporosis can significantly impact the quality of life, resulting in disability and increased mortality.^1^ Osteoporotic fractures are thought to initiate disability or vulnerability, followed by a progression to death. Although these fractures may not directly cause death, individuals with fractures exhibit a higher mortality rate than those without fractures.^2^^,^^3^ During the phase between the fracture and death, frailty in the fracture victim probably increases. Frailty due to decreased physical strength leads to a higher risk of falling, leading to fractures. Conversely, disability resulting from fractures can also increase frailty. Thus, fractures and frailty are considered to have a reciprocal relationship.^1^ This suggests the presence of common underlying health factors that may contribute to the progression of frailty and osteoporotic fractures. Therefore, it is crucial not only to concentrate on fracture prevention but also to address these concealed health issues in the management and healthcare of osteoporosis patients.

Fractures of representative osteoporosis include proximal femoral fractures, distal radius fractures, proximal end of humerus (surgical neck) fractures, and vertebral fractures. Among these, vertebral fractures are the most frequently observed. Once they occur, the fractured vertebra remains deformed, often resulting in changes in posture.^4^ Additionally, height loss due to vertebral fractures leads to a decrease in the volume of body cavities, resulting in functional abnormalities of visceral organs within these cavities.^5^ While most proximal femoral fractures and distal radius fractures are almost fully restored through surgeries, vertebral fractures often leave behind significant functional impairments. Therefore, prevention is extremely important. With recent advances in osteoporosis pharmacotherapy, more than half of vertebral fractures have become preventable.^6^ However, only 20% of osteoporosis cases currently receive treatment.^6^ Therefore, many patients still suffer from disability due to vertebral fractures. Elucidating the pathophysiology of vertebral fractures caused by osteoporosis is extremely important.

Individuals with osteoporosis frequently exhibit heightened vulnerability to their overall health, manifested as increased frailty.^1^ Osteoporosis shares common phenotypes with frailty, including muscle weakness^7^^,^^8^ and unintentional weight loss,^9^ both contributing to an elevated risk of fractures.^10^^,^^11^ This condition also increases self-reported exhaustion, weakness, slow walking speed, and low physical activity.^12^ The Cardiovascular Health Study (CHS) Index is often used to evaluate frailty. This index consists of a questionnaire related to 5 items: exhaustion, muscle weakness, slowness, low activity, and weight loss.^13^ The FRAIL-J Scale was developed to be suitable for Japanese frailty assessment. This scale consists of a questionnaire that explores 5 items: fatigue, resistance, ambulation, inactivity, and weight loss. The strong association between frailty and the risk of falls, fractures, and mortality in older women was emphasized by Ensrud et al.^14^ We have also reported that osteoporosis is linked to the progression of frailty.^15^

Various biochemical parameters closely associated with both bone fractures and frailty have been identified. Notably, interleukin-6 (IL-6), an inflammatory cytokine, emerges as a potential link between osteoporotic fractures and frailty. Independent studies by Cauley et al.^16^ and Barbour et al.^17^^,^^18^ reported that elevated serum levels of IL-6 independently contribute to the risk of incident fractures across diverse populations. Conversely, IL-6 produced by muscles during exercise plays a role in regulating glucose homeostasis and mediating lipolysis.^19^ Muscles release IL-6 in response to energy deficit, facilitating somatic energy liberation through lipolysis—a process referred to as energy allocation.^20^ Therefore, a high serum level of IL-6 may signify insufficient muscular energy. Indeed, frail older adults exhibit higher serum levels of IL-6, correlating with lower muscle power and performance.^21^

Adiponectin is the other candidate of the common pathophysiological element among osteoporotic fractures and frailty. We demonstrated that adipokines, including adiponectin and leptin, are independent predictors for osteoporotic fractures in different bone sites.^22^ Nagasawa et al.^23^ reported that higher plasma adiponectin levels were associated with frailty.

Considering the similarity in phenotypes between osteoporotic fractures and frailty, our hypothesis is that osteoporosis and frailty share biological backgrounds. The accumulating evidence suggests that IL-6 or adiponectin may serve as core factor(s) contributing to both osteoporotic fractures and frailty. Inflammatory cytokines are known risk factors for fractures,^16–18^ and adipokines have also been identified as contributors to fracture risk.^24^ Adiponectin and IL-6 are recognized as risk factors for sarcopenia and frailty.^25^ However, to the best of our knowledge, there are no data confirming whether IL-6 and adiponectin are risk factors for frailty and osteoporotic fractures in the same population and in a Japanese population. The objective of this study is to examine how IL-6 and adiponectin are involved in the pathophysiology of frailty assessed by the FRAIL-J Scale and osteoporotic vertebral fractures in a cohort of Japanese older women.

## Materials and methods

### Participants

The participants in this analysis were selected from the Nagano Cohort Study, which is an ongoing registration study comprising peri- or postmenopausal female outpatients visiting a primary care institute in Nagano Prefecture, Japan since 1993.^3^^,^^22^^,^^26^^,^^27^ The study was conducted in accordance with the principles outlined in the Declaration of Helsinki. All participants provided comprehensive written informed consent, and they were observed according to a prespecified protocol that was reviewed and approved by the ethics committee of the institution. A total of 2284 participants were registered from 1993 to 2023. Among them, 1171 participants had their frailty status assessed. They were all capable of independent walking at the baseline. Participants who met exclusion criteria, such as critical illness (eg, acute serious infectious disease), terminal stage malignancy, or long-term steroid use, were excluded from the study. Additionally, individuals with end-stage chronic renal failure with uremia, estimated glomerular filtration rate of less than 20 mL/min/1.73 m,^2^ or collagen disease were also excluded. Subjects with primary hyperparathyroidism, vitamin D deficiency [defined as serum 25(OH) vitamin D levels less than 10 ng/mL], postpartum osteoporosis, or anorexia nervosa were also excluded. After eliminating subjects who met these exclusion criteria (*n* = 159), 912 participants were included in this study. The study follow-up was concluded when participants passed away or discontinued follow-up due to relocation or institutionalization. Since the primary endpoint of this study was the occurrence of vertebral fractures, the follow-up period was terminated at the time of a vertebral fracture.

### Measurements

At the baseline examination, body weight and height were measured. Grip strength was measured at baseline with a dynamometer (TTM, Chiba, Japan). Each participant’s body weight was tracked until the conclusion of the study. DXA (DPX series, GE Healthcare Lunar, Madison, WI) was used to determine BMD at the lumbar spine (L_2-4_) and hips (total hip). The fat mass at the trunk and hip was simultaneously measured by DXA. Serum and urine samples were collected under non-fasting conditions to measure biochemical markers.

We also measured the associations of IL-6 and adiponectin with the other biochemical indices, which have been reported to be risk factors for incident fractures or frailty, including urinary excretion of NTx (Osteomark; Creative Diagnostics, NY, United States) for bone resorption. Serum homocysteine and urinary excretion of pentosidine were measured as risk factors for tissue degeneration and bone fractures. Urinary excretion of pentosidine was measured by the ELISA kit^28^ and standardized by the urinary creatinine concentration. The serum level of homocysteine was measured by an enzymatic method (HPLC method, LSI Medience, Tokyo, Japan). Serum levels of sclerostin and FGF 23 were measured by specific ELISA Kits (Sclerostin ELISA kit, Life Technologies Co., CA, United States, and Cynos FGF-23 ELISA kit, Cynos Co., Tokyo, Japan) for evaluating osteocytic function. Urinary excretion of NTx (Osteomark; Creative Diagnostics, NY, United States) was measured.

Serum calcium and phosphate levels were measured in-house using dry chemistry methods (Fuji dry chemistry Co., Tokyo, Japan). Serum interleukin-6 (IL-6) levels were determined with an ELISA kit (QuantiGlo Human IL-6 ELISA Kit; Bio-Techne Co., Minneapolis, MN, United States). The other serum biochemical markers were measured at LSI Medience (Tokyo, Japan).

### Fracture assessment

Prevalent vertebral fractures were diagnosed by semiquantitative analysis using X-ray films of the thoracic and lumbar spines with grade 1 or higher deformities (ie, more than 10% reduction in vertebral body area) at baseline.^29^ The judgment of incident vertebral fractures was made by at least two well-trained physicians, and the diagnosis of incident vertebral fractures was validated by the criteria proposed by Genant et al.^30^ and referred to our database of vertebral height measurement.^29^

### Assessment of frailty

The FRAIL-J Scale was adapted from the original version, considering the social and cultural customs specific to Japan. Comparable existing items were utilized for this modification.^13^ Fatigue, resistance, and weight loss were obtained from the Kihon Checklist, which was developed by the Japanese Ministry of Health, Labor, and Welfare and is widely used to identify older adults at risk of requiring long-term care. Ambulation was derived from the Kaigoyobo Checklist, another well-established index for assessing the risk of long-term care. The questionnaire contained yes/no questions that focused on 5 items: (1) fatigue: in the last 2 wk, have you felt tired without a reason? 1 = Yes, 0 = No; (2) resistance: do you normally climb stairs without using the handrails or the wall for support? 0 = Yes, 1 = No; (3) ambulation: by yourself and not using aids, do you have any difficulty walking 1 km without resting? 1 = Yes, 0 = No; (4) inactivity: does your sitting or lying time account for 80% or more of your waking time in a day? 1 = Yes, 0 = No; (5) weight loss: have you had unintentional weight loss >2-3 kg in the past 6 mo? 1 = Yes, 0 = No. Frailty levels were assessed using frailty scores ranging from 0 to 5. In a cross-sectional analysis, frailty status in the participants was categorized into 3 groups: frail group (frail score 3 and above), pre-frail group (frail score 1 and 2), and not frail group (frail score 0).

### Statistical analysis

The measured baseline characteristics were presented as mean ± SD or proportion (%), and the statistical difference between the group with and without incident vertebral fracture was assessed using ANOVA or χ^2^ test. A Cox proportional hazards model was used to estimate the hazard ratio (HR) and 95% CIs of incident vertebral fracture. If a participant transferred to another hospital or died during the observation period, they were judged as censored in the Cox model. For continuous explanatory variables, the range of increase was defined and the HR for each increase was calculated. Candidate factors for the Cox model from baseline measurements were selected from those that were significantly different (*p*<.05) between groups with and without incident vertebral fracture, and these factors were extracted using the stepwise method. The Kaplan-Meier curves were plotted to illustrate the survival curve of the incident vertebral fractures among the quarter groups of IL-6 concentrations during the observation period, and a log-rank test was used to assess the statistical significance. The odds ratio (OR) and 95% CIs between frailty status and incident vertebral fracture were evaluated by logistic regression analysis. The association between the frailty status at the time of registration and baseline characteristics was evaluated using multivariate logistic analysis. To elucidate the factors independently associated with IL-6 or adiponectin, the items that demonstrated significant correlation with IL-6 or adiponectin were incorporated into multivariate regression analysis for IL-6 or adiponectin as a dependent variable and various items as explanatory variables.

All comparisons were 2-sided, and *p* values <.05 were considered statistically significant. Data were analyzed using JMP version 16.0 (SAS Institute, Cary, NC, United States).

## Results

A total of 912 participants who were evaluated for frailty, with a mean age of 63.9 ± 10.0 yr, were followed up for an average duration of 10.5 ± 7.5 yr. However, only 876 participants were followed up for incident vertebral fractures. Table S1 presents the background data of the participants with or without incident vertebral fractures during the observation period. Subjects with a fracture were older and exhibited higher serum adiponectin and log IL-6 levels than the non-fracture group. Additionally, higher urinary excretion of NTx and pentosidine was observed in the fracture group compared with the non-fracture group. Conversely, the fracture group showed significantly lower grip strength, L2-4 bone mineral density (LBMD), total hip BMD, serum leptin levels, cCa, and PTH than the non-fracture group. The prevalence of prior vertebral fractures in the fracture group was 33.2%, whereas the non-fracture group had only 12.0% (*p*<.001). The fracture group indicated a significantly higher prevalence of frailty (46.5%) than the non-fracture group (17.5%) (*p*<.0001).

To select the independent risk of incident vertebral fracture, stepwise regression analysis was performed using variables that demonstrated significant differences between the groups with or without incident vertebral fractures, as presented in Table S1.

As indicated in Table 1, the LBMD, cCa, PTH, adiponectin, IL-6, presence of prevalent vertebral fractures, and frailty status exhibited significant association with incident vertebral fractures after adjustment of confounders. Increased LBMD, cCa, and PTH were negatively associated with vertebral fractures, while increased adiponectin and IL-6 positively contributed to the incident vertebral fractures. Furthermore, the presence of vertebral fractures and frailty positively contributed to incident vertebral fractures.

**Table 1:** Cox proportional hazard model in variables showing significant association with incident vertebral fractures.

**Variable**	**HR**	**95% CI**		***p***
**LBMD, 0.1 g/cm** ^**2**^ **up**	0.681	0.592	0.783	<.001
**cCa, 1 mg/dL up**	0.588	0.416	0.831	.003
**PTH, 1 pg/mL up**	0.986	0.973	0.999	.025
**Adiponectin, 1** μ**g/mL up**	1.039	1.012	1.067	.004
**Log IL-6 (pg/mL), 1 up**	1.413	1.100	1.814	.007
**OP treatment, yes**	0.837	0.526	1.331	.451
**Prevalent vertebral fracture, yes**	2.002	1.220	3.288	.007
**Frail, frail/pre-frail/no frail**	1.839	1.432	2.361	<.001

“up” indicates a unit increase in each explanatory variable, and the HR values calculated with each increase were shown. Abbreviations: HR, hazard ratio; LBMD, lumbar (L2-4) bone mineral density, prevalent fracture included prevalent vertebral fractures in the T4 to L4 vertebral column; log IL-6, log-transformed interleukin-6 levels in serum; OP, outpatient.

**Figure 1:**
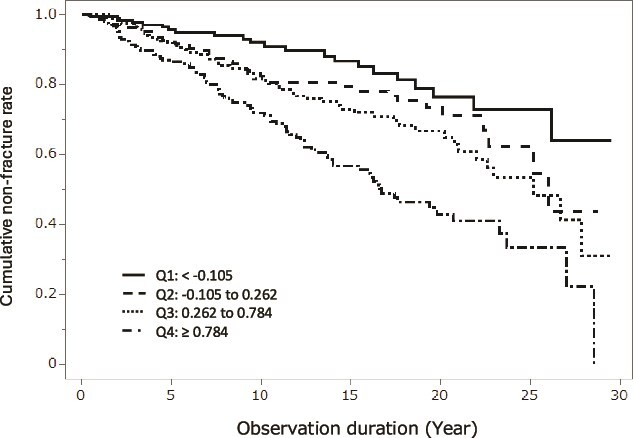
Incident vertebral fracture rate in the quartiles of serum log-transformed IL-6 levels. Ranges of quartiles of serum log-transformed IL-6 (pg/mL) were Q1: < −0.105, Q2: −0.105 to 0.262, Q3: 0.262 to 0.784, Q4: ≥ 0.784.

Figure 1 illustrates the relationship between quartiles of serum log IL-6 levels and the cumulative non-fracture rate.

Figure 1 illustrates the relationship between quartiles of serum log IL-6 levels and the cumulative non-fracture rate. Subjects in the highest quartile of IL-6 (Q4) exhibited a faster and higher incident vertebral fracture rate than the other quartiles (*p*<.001 by the log-rank test).

**Figure 2:**
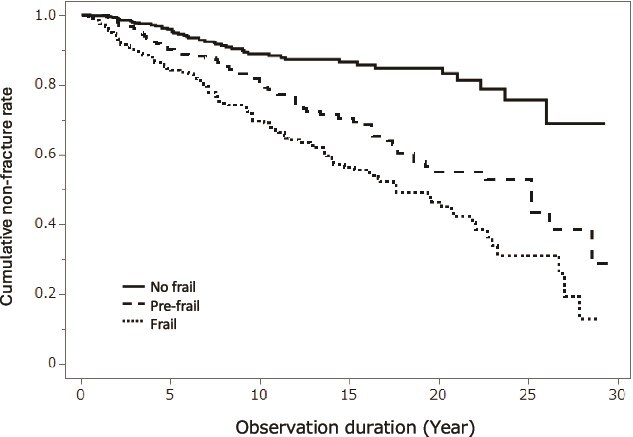
Incident vertebral fracture rate in the baseline status of frailty.

To investigate the relationship between the status of frailty at registration and the future occurrence of vertebral fractures, the time course of vertebral fracture occurrence in each category was evaluated using Kaplan-Meier analysis. Frailty status significantly contributed to the incidence of vertebral fractures. (Figure 2, No frail vs Frail: *p*<.001 by the log-rank test). The ORs and their 95% CIs between each group are shown in Table 2. Significant differences were observed in all combinations among the 3 frailty groups, indicating an association between frailty status and fracture occurrence, including in pre-frailty status.

**Table 2:** Odds ratio for incident vertebral fractures by the baseline frailty.

**Group**	**OR**	**95% CI**		***p***
**Pre-frail versus not frail**	3.60	2.36	5.54	<.001
**Frail versus not frail**	7.20	4.77	10.92	<.001
**Frail versus pre-frail**	1.99	1.34	2.97	.006

Abbreviation: OR, odds ratio.

Subsequently, the study examined whether the fracture risk factors listed in Table 1 were associated with the frailty status at the time of registration using multivariate logistic regression analysis. As shown in Table 3, factors between frailty status and factors associated with vertebral fracture risk were adiponectin, IL-6, and prevalent vertebral fractures. However, LBMD and the cCa level were not associated with frailty status.

**Table 3:** The association of fracture risks with frailty status using multivariate logistic regression analysis.

**Variable**	**OR**	**95% CI**		***p***
**LBMD, 0.1 g/cm** ^**2**^ **up**	0.917	0.825	1.018	.101
**cCa, 1 mg/dL up**	0.755	0.541	1.054	.103
**Adiponectin, 1 μg/mL up**	1.063	1.037	1.091	<.001
**Log IL-6 (pg/mL), 1 up**	2.294	1.804	2.916	<.001
**Prevalent vertebral fracture, yes**	2.816	1.784	4.446	<.001

“up” indicate a unit increase in each explanatory variable, and the OR values calculated with each increase were shown. Abbreviations: OR, odds ratio; LBMD, lumbar (L2-4) bone mineral density; log IL-6, log-transformed interleukin-6 levels in serum.

These results suggest a reciprocal relationship between frailty and incident vertebral fractures. Frailty appears to trigger the occurrence of vertebral fractures, while existing vertebral fractures exacerbate the degree of frailty. Additionally, the biochemical factors IL-6 and adiponectin were identified as common associators of the underlying pathophysiology in both frailty and vertebral fractures.

These findings prompted an examination of the associations between IL-6 and adiponectin with various metabolic markers. Therefore, we investigated whether log IL-6 and adiponectin levels were associated with any metabolic parameters. The results are shown in Table S2. Since log IL-6 and adiponectin demonstrated significant correlations with various metabolic markers, we also conducted a multivariate analysis using log IL-6 and adiponectin levels as dependent variables, along with the metabolic markers associated with them as explanatory variables (Table 4). The results indicated that log IL-6 was significantly associated with low grip strength, Log CRP, FGF 23, and fat tissue mass around the hip joint. Conversely, adiponectin was significantly associated with femoral bone density, homocysteine, trunk fat mass, grip strength, and PTH. These findings indicated that both IL-6 and adiponectin are common risk factors for fracture occurrence and frailty; however, the metabolic factors associated with them are different.

**Table 4:** Multivariate analysis for serum levels of log IL-6 and adiponectin.

**Log IL-6**					**Adiponectin**				
**Item**	**PE**	**SE**	***t***	***p***	**Item**	**PE**	**SE**	***t***	***p***
**Intercept**	0.83	0.30	2.75	.0061	Intercept	28.76	2.89	9.95	<.0001
**Grip strength**	−0.05	0.01	−6.82	<.0001	HBMD	−6.93	2.43	−2.86	.0045
**Homocysteine**	0.00	0.01	−0.01	.0060	NTx	−0.01	0.01	−0.58	.5627
**Pentosidine**	0.00	0.00	1.36	.1733	Log CRP	0.22	0.27	0.82	.4120
**Log CRP**	0.11	0.03	3.88	.0001	Pentosidine	0.02	0.02	0.94	.3477
**FGF23**	0.01	0.002	2.28	.0232	Homocysteine	0.37	0.09	3.92	.0001
**Sclerostin**	0.00	0.00	1.53	.1275	Trunk fat mass	−0.28	0.03	−0.16	<.0001
**Hip fat mass**	0.02	0.01	2.43	.0155					

To elucidate the factors independently associated with IL-6 or adiponectin, the items that demonstrated significant correlations with IL-6 or adiponectin were incorporated into multivariate regression analysis for IL-6 or adiponectin as a dependent variable and various items as explanatory variables. Abbreviation: Log IL-6, log-transformed interleukin-6 levels in serum.

## Discussion

Frailty is considered to be the state in which a decrease in overall physical activity occurs primarily through a decrease in muscle activity, accompanied by sarcopenia, resulting in malnutrition, and weight loss. These processes are believed to create a kind of vicious cycle, further progressing sarcopenia. We have previously reported^14^ that the presence of osteoporosis contributes to the progression of frailty. Frailty is understood to be a condition in which various physiological functions decline due to deterioration in overall metabolic status in elderly individuals, leading to a loss of independence in daily life, necessitating care, and ultimately leading to death.^31^ Osteoporosis itself is not a direct cause of death but is known to increase the mortality rate, due to vulnerability caused by fractures. Therefore, osteoporosis and frailty can be considered diseases that raise mortality rates, as they induce vulnerability in bones and muscles, directly or indirectly, even if they may not directly cause death. Frailty and osteoporosis are known to mutually exacerbate each other, and a close relationship between osteoporosis and frailty is assumed.^1^^,^^15^ In this study, we examined whether there are any common factors between the occurrence of osteoporotic fractures and the presence of frailty.

First, we examined the factors contributing to incident vertebral fractures. Numerous factors with significant differences between the participants with or without incident vertebral fractures were observed, as shown in Table S1. Among these, factors determined by stepwise regression were selected (Table 1), including LBMD, prevalent osteoporotic fractures, log-transformed IL-6, presence of frailty, cCa level, PTH concentration, and adiponectin level. After 2000, bisphosphonates were introduced into the Japanese market, and the majority of registered participants in the Nagano cohort study were treated with these drugs. These treated participants were also given vitamin D preparations at the baseline. As a result, the serum calcium level was higher in treated patients than those without treatment. This difference in serum calcium levels may be why the OR of corrected calcium for incident vertebral fractures was lower in the treated group than in the untreated group. Additionally, the serum level of adiponectin was found to be a significant risk factor for incident vertebral fractures, which aligns with our previous reports.^22^

When considering the time dependency in Cox regression by including these factors, the predictors for incident fracture were LBMD, prevalent fractures, frailty status, cCa level, adiponectin, and log IL-6 (Table 3). Since IL-6 has been reported to be associated with fracture occurrence^16–18^ and frailty presence,^21^ these results were understandable. However, this report is the first to investigate both frailty and fracture occurrence in the same population, confirming the effect of IL-6 and adiponectin shared with osteoporotic fracture and frailty. Serum IL-6 levels were found to be higher in the fracture group. Serum level of IL-6 was a significant independent risk factor for vertebral fractures in this study (Table 3), consistent with previous reports.^16–18^

Frailty has been identified as a significant risk factor for incident vertebral fractures. Therefore, we investigated the factors associated with acquiring frailty using logistic regression analysis. As shown in Table 3, the results revealed a correlation between the presence or absence of frailty and adiponectin, IL-6, and prevalent vertebral fractures. This suggests a close association between frailty and the risk of fractures. In our study population, grip strength, a phenotype of frailty, demonstrated a strong negative correlation with IL-6 (*r* = −0.333, *p*<.0001) (Figure S2). Additionally, grip strength exhibited a significant negative correlation with serum levels of adiponectin (Figure S3) (*R* = −0.195, *p*<.000). Although grip strength showed significant differences between the presence and absence of vertebral fractures (refer to Table S1), it was not considered a predictor of fractures. This is likely due to its strong confounding relationship with IL-6 or adiponectin. Low grip strength and high IL-6 levels were both data recorded at the time of study entry. Thus, it is difficult to determine whether low grip strength causes high IL-6 levels inducing fractures or whether high IL-6 levels cause low grip strength inducing fractures. However, low grip strength is one of the diagnostic criteria for sarcopenia, and patients with sarcopenia are known to be at high risk for fractures. Therefore, low grip strength could itself be an important factor in the development of fractures.

The data present here strongly suggested that IL-6 and adiponectin were risk factors for incident vertebral fractures and frailty simultaneously. Ma et al.^21^ reported that elevated serum IL-6 and adiponectin levels are associated with frailty in a small case-control cross-sectional study (*n* = 130). Our results were consistent with the previous study.

This study and previous reports indicate that IL-6 and adiponectin may play significant roles in the incidence of osteoporotic fractures and frailty. Identifying the parameters that regulate or are associated with these 2 cytokines is necessary. Thus, we explored the parameters associated with IL-6 and adiponectin levels. As shown in Table S2, various parameters were linked to these cytokines. Multivariate analysis revealed that IL-6 levels were independently associated with grip strength, homocysteine, CRP, FGF23, and fat mass around the hips. In contrast, adiponectin levels were independently associated with hip BMD, homocysteine, and visceral fat mass (Table 4).

The association with homocysteine was common to both IL-6 and adiponectin serum levels. Homocysteine, an intermediate metabolite in the methionine cycle, is known to induce tissue protein degeneration, leading to fractures and atherosclerosis.^32^ Fat mass was significantly associated with both IL-6 and adiponectin, but the location of fat mass and the direction of the correlations differed between them. Accumulation of fat mass in the trunk area was associated with lower adiponectin levels, while fat mass in the hip region was associated with higher IL-6 levels. Additionally, IL-6 levels were associated with CRP, suggesting that IL-6 may be influenced by chronic inflammation. IL-6 was linked to muscle strength, whereas adiponectin was associated with BMD and muscle strength. IL-6 levels were significantly associated with FGF23 level (*p*=.0232). This evidence may indicate that IL-6 is linked to osteocyte function (FGF23),^33^ muscle function, tissue degeneration, and inflammation.

On the other hand, adiponectin is linked with BMD, tissue degeneration, and fat mass. This evidence may indicate that IL-6 and adiponectin are common risk factors for fractures and frailty. However, the factors associated with IL-6 and adiponectin were different, suggesting that these 2 common factors may be regulated by different metabolic factors.

Adiponectin and IL-6 were closely related to various factors, all of which are suggested to contribute to the occurrence of fractures and the acquisition of frailty. Therefore, IL-6 and adiponectin are common risk factors underlying the occurrence of fractures and the acquisition of frailty, but their contributions to each condition probably differ. In other words, fractures and frailty are linked to each other, but the 2 cytokines that serve as common risk factors are anticipated to be involved through different mechanisms.

This study has several limitations that should be considered. First, the studied population was relatively small and limited to Japanese women. Therefore, a future study would be desirable to include the general population and other ethnic groups. Additionally, this study was conducted in a subset of the Nagano cohort study population, which may introduce selection bias. However, the baseline data of the current study were similar to those previously reported in terms of participant age, body composition, and BMD.^15^^,^^22^^,^^26–28^ Furthermore, the background data are comparable with those of the general Japanese population.^27^ In addition, the participants visited a primary care institution to undergo treatment for their general health problems. However, the presence of comorbidities in the participants was nearly equal to those in the general population of community-dwelling older Japanese women.^27^ As such, the risk of selection bias in the present population was low. Another limitation is that some patients were treated for osteoporosis for ethical reasons, which may lead to an underestimation of the incidence of vertebral fractures.

While acknowledging the limitations of this study, we can conclude that there is a close relationship between frailty and osteoporotic fractures, mediated through the metabolisms of interleukin-6 (an inflammatory cytokine) and adiponectin (an adipokine). These findings offer new insights for managing frailty and osteoporosis in the future.

## Supplementary Material

SuppleTable1JBMRPLUSDec21st_ziaf009

SuppleTable2_ziaf009

SupleFig1JBMRPLUS_ziaf009

SupleFig2JBMRPLUS_ziaf009

SupleFig3JBMRPLUS_ziaf009

## Data Availability

The data that support the findings of this study are available from the corresponding author, upon reasonable request.
