# Supplementary material for: Frailty and osteoporotic fractures represent mutual risks for each other with common physiological backgrounds
Source: JBMR Plus. 2025 Jan 13;9(4):ziaf009. doi: 10.1093/jbmrpl/ziaf009 (PMC11886566; doi:10.1093/jbmrpl/ziaf009)
Supplement: SuppleTable1JBMRPLUSDec21st_ziaf009 [file suppletable1jbmrplusdec21st_ziaf009.docx]

***Supplemental Table 1.* Background data of the participants with or without incident vertebral fractures.**

| Variables | Incident vertebral fracture | | P |
| --- | --- | --- | --- |
|  | No (n = 674) | Yes (n = 202) |  |
| Age in years | 63.0±10.1 | 66.1±8.2 | < 0.001 |
| BMI, Kg/m^2^ | 22.3±3.2 | 22.5±3.2 | 0.389 |
| LBMD, g/cm^2^ | 0.988±0.190 | 0.809±0.167 | < 0.001 |
| HBMD, g/cm^2^ | 0.787±0.126 | 0.741±0.115 | < 0.001 |
| cCa, mg/dL | 9.1±0.6 | 9.0±0.5 | 0.018 |
| P, mg/dL | 3.5±0.5 | 3.5±0.5 | 0.986 |
| PTH, pg/mL | 41.8±15.5 | 38.9±18.7 | 0.029 |
| 25OHVD, ng/mL | 20.0±6.1 | 20.6±5.9 | 0.223 |
| NTx, nMBCA/mMCr | 49.0±24.7 | 54.9±28.2 | 0.004 |
| Adiponectin, μg/mL | 14.3±6.8 | 17.2±8.8 | < 0.001 |
| Leptin, ng/mL | 10.7±8.4 | 9.1±8.8 | 0.015 |
| Log hCRP, mg/dL | -2.99±1.14 | -2.90±1.08 | 0.338 |
| Pentosidine, pM/mgCr | 34.0±14.3 | 40.7±26.8 | < 0.001 |
| Log IL-6, pg/mL | 0.32±0.77 | 0.73±0.82 | < 0.001 |
| Grip strngth, Kg | 19.8±4.3 | 17.7±4.5 | < 0.001 |
| Observation duration, years | 10.7±7.6 | 9.6±6.9 | 0.066 |
| Treatment of osteoporosis, Yes% | 43.5% | 60.4% | < 0.001 |
| Prevalent vertebral fracture, Yes% | 12.0% | 33.2% | < 0.001 |
| Frail, Yes% | 17.5% | 46.5% | < 0.001 |

*LBMD: Lumbar spine bone mineral density (L), HBMD: Total hip bone mineral density, cCa: Serum calcium level corrected by serum albumin, P: Phosphate, PTH: Parathyroid hormone, 25OHVD: 25-hydroxycholecalciferol, NTx: type I collagen cross-linked N-telopeptides, Log hCRP: Log-transformed high-sensitive C-reacting protein, Log IL-6: Log-transformed Interleukin-6.*
