# Supplementary material for: Frailty and osteoporotic fractures represent mutual risks for each other with common physiological backgrounds
Source: JBMR Plus. 2025 Jan 13;9(4):ziaf009. doi: 10.1093/jbmrpl/ziaf009 (PMC11886566; doi:10.1093/jbmrpl/ziaf009)
Supplement: SuppleTable2_ziaf009 [file suppletable2_ziaf009.docx]

***Supplemental Table 2.*** Correlation coefficients of Log IL-6 and Adiponectin with various markers.

| Tissues | Markers | For Log IL-6 | | | |  | For adiponectin | | | |
| --- | --- | --- | --- | --- | --- | --- | --- | --- | --- | --- |
|  |  | R | P | N | Direction |  | R | P | N | Direction |
| Bone | LBMD | 0.00 | NS | 871 | NA |  | 0.12 | 0.0007 | 807 | (-) |
|  | HBMD | 0.03 | NS | 860 | NA |  | 0.27 | <0.0001 | 797 | (-) |
|  | NTx | 0.00 | NS | 858 | NA |  | 0.14 | 0.0003 | 727 | (+) |
|  | Sclerostin | 0.11 | 0.004 | 724 | (+) |  | 0.00 | NS | 732 | NA |
|  | FGF 23 | 0.19 | <0.0001 | 715 | (+) |  | 0.03 | NS | 734 | NA |
| Inflammation | Log hCRP | 0.25 | <0.0001 | 838 | (+) |  | 0.12 | 0.0008 | 795 | (-) |
|  | Log IL-6 | NA | NA | NA | NA |  | 0.10 | 0.0095 | 778 | (+) |
| Matrix protein degeneration | Pentosidine | 0.14 | 0.0001 | 728 | (+) |  | 0.13 | 0.0002 | 675 | (+) |
|  | Homocysteine | 0.13 | 0.0002 | 858 | (+) |  | 0.13 | 0.0002 | 806 | (+) |
| Adipose tissue | Trunk fat mass | 0.12 | 0.0005 | 861 | (+) |  | 0.424 | <0.0001 | 795 | (-) |
|  | Hip fat mass | 0.17 | <0.0001 | 848 | (+) |  | 0.06 | NS | 793 | NA |
|  | Leptin | 0.03 | NS | 800 | NA |  | 0.22 | <0.0001 | 796 | (-) |
|  | Adiponectin | 0.10 | 0.0095 | 778 | (+) |  | NA | NA | NA | NA |
| Muscle | Grip strength | 0.33 | <0.0001 | 834 | (-) |  | 0.20 | 0.0001 | 760 | (-) |
| Calcium metabolism | PTH, pg/ml | 0.00 | NS | 831 | NA |  | 0.14 | <0.0001 | 726 | (+) |
|  | 25(OH)D,ng/ml | 0.00 | NS | 803 | NA |  | 0.00 | NS | 778 | NA |
|  | cCa, mg/dl | 0.00 | NS | 799 | NA |  | 0.00 | NS | 726 | NA |

R: regression coefficient, P: Level of significance, N: Number of cases, LBMD: Lumbar spine bone mineral density (L), HBMD: Total hip bone mineral density, cCa: Serum calcium level corrected by serum albumin, PTH: Parathyroid hormone, 25OHVD: 25-hydroxycholecalciferol, NTx: type I collagen cross-linked N-telopeptides, Log hCRP: Log-transformed high-sensitive C-reacting protein, Log IL-6: Log-transformed Interleukin-6.

Trunk and Hip fat mass were measured by DXA at L2-4 and total hip region, respectively. Grip strength was measured by two time-trials at right and left side grips, and the maximum performance was taken as a representative value. The term "direction" indicates whether the correlation attitude is positive or negative. NA means not applicable.
