# Supplementary material for: Frailty and osteoporotic fractures represent mutual risks for each other with common physiological backgrounds
Source: JBMR Plus. 2025 Jan 13;9(4):ziaf009. doi: 10.1093/jbmrpl/ziaf009 (PMC11886566; doi:10.1093/jbmrpl/ziaf009)

Supplemental Figure 1. Correlation between urinary excretion of pentosidine and serum IL-6 log transformed.

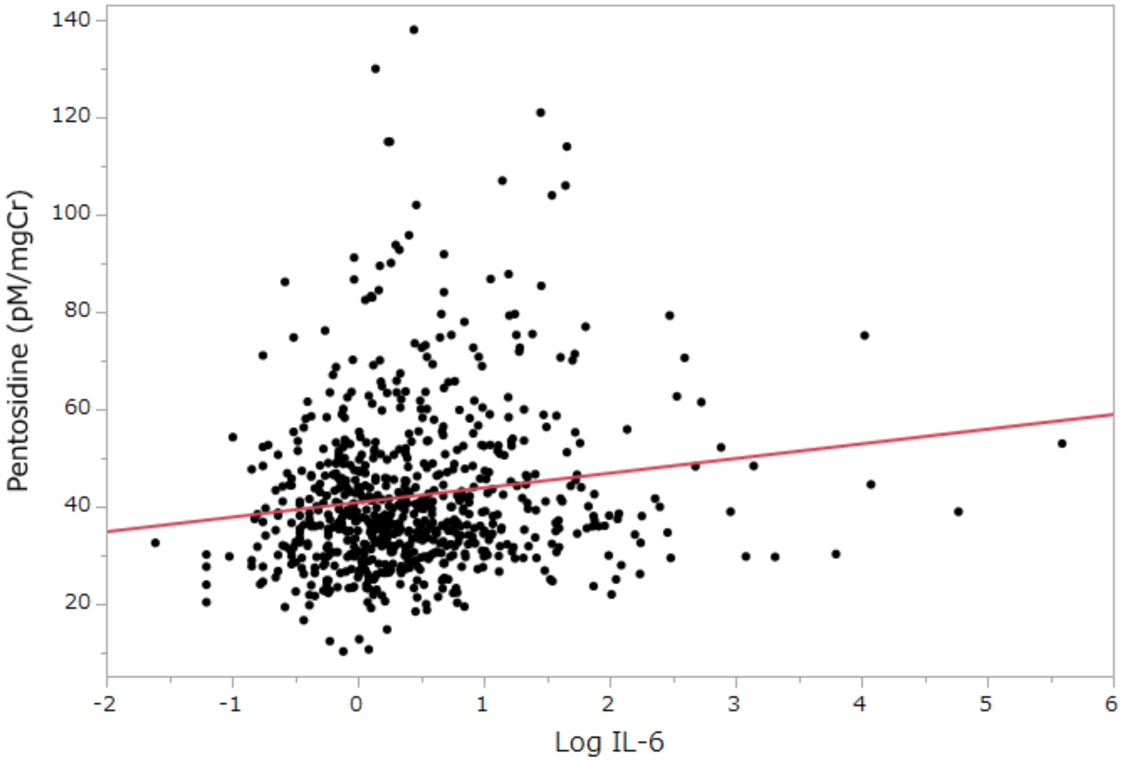

Supplement: SupleFig1JBMRPLUS_ziaf009 [file suplefig1jbmrplus_ziaf009.pdf]
