# Supplementary figures and images for: Frailty and osteoporotic fractures represent mutual risks for each other with common physiological backgrounds
Source: JBMR Plus. 2025 Jan 13;9(4):ziaf009. doi: 10.1093/jbmrpl/ziaf009 (PMC11886566; doi:10.1093/jbmrpl/ziaf009)

**Supplemental Figure 2.** Relationship between grip strength and serum IL-6 log transformed.

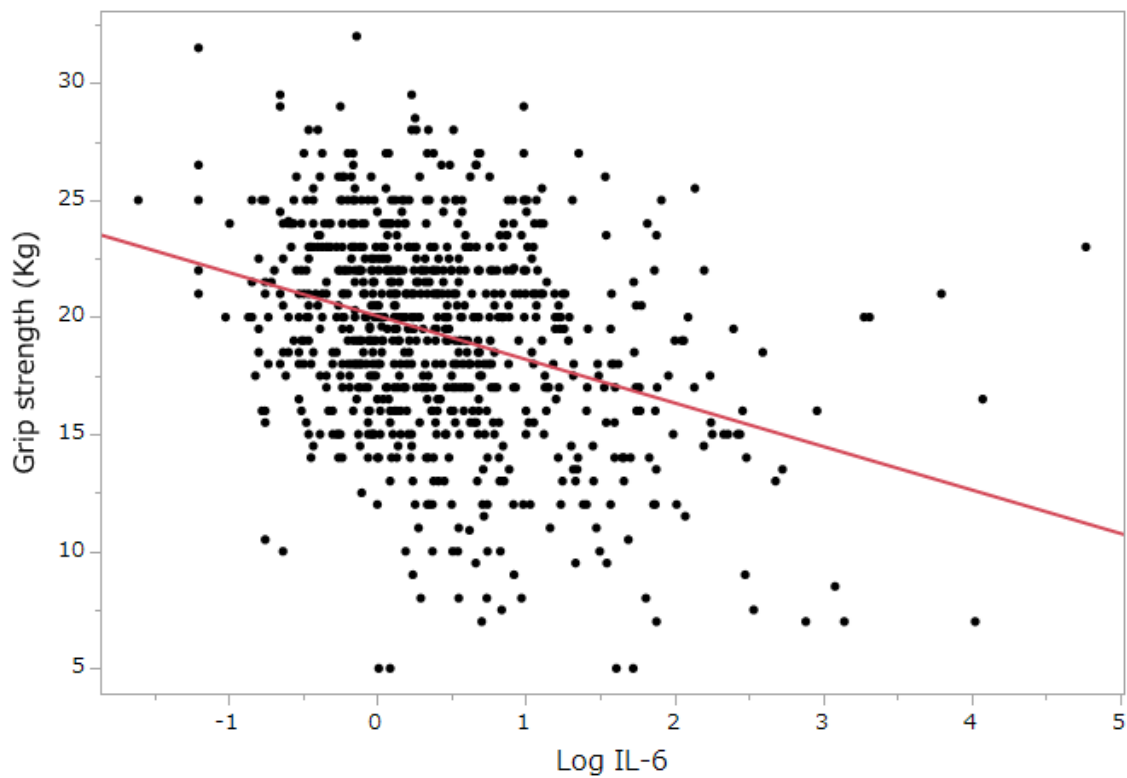

Supplement: SupleFig2JBMRPLUS_ziaf009 [file suplefig2jbmrplus_ziaf009.pdf]

**Supplemental Figure 3.** Relationship between grip strength and serum adiponectin levels.

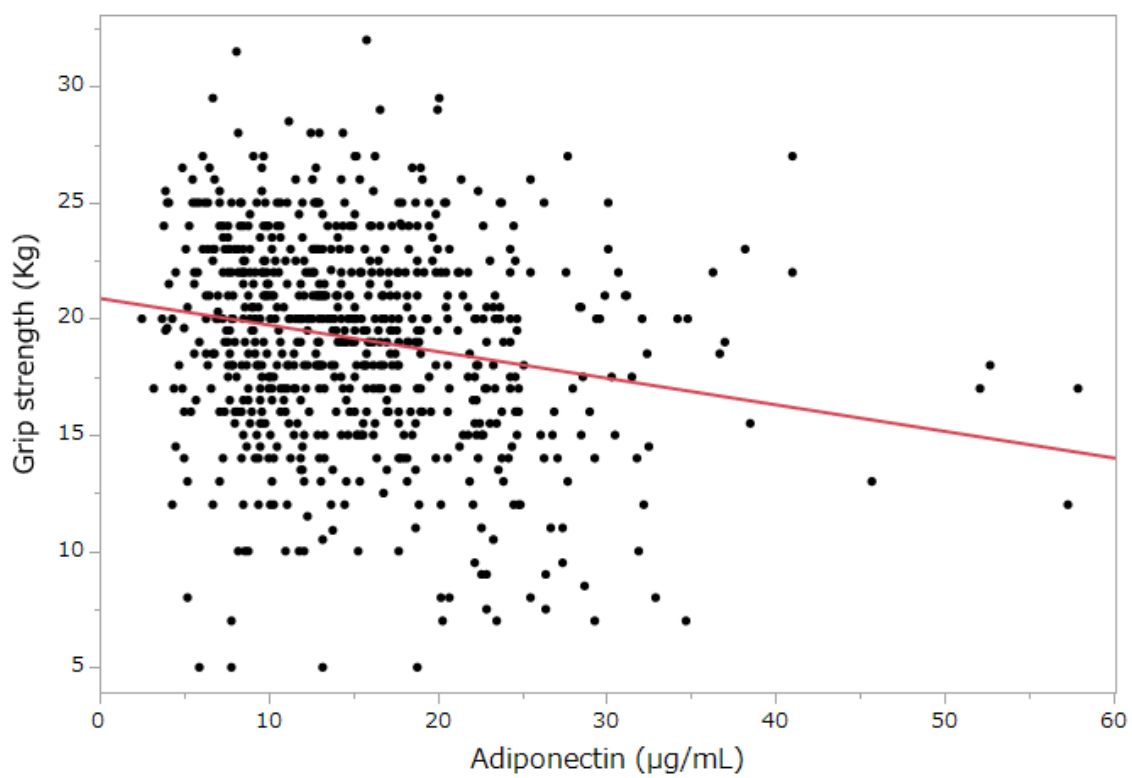

Supplement: SupleFig3JBMRPLUS_ziaf009 [file suplefig3jbmrplus_ziaf009.pdf]
